# Lactate Uptake by MCT4 Facilitates Stability and Suppressive Function of Tumor-Infiltrating Regulatory T Cells by Promoting Foxp3 Lactylation

**DOI:** 10.3390/ijms27104619

**Published:** 2026-05-21

**Authors:** Zhaofei Wu, Yuwei Liu, Wei Xian, Jingyi Wang, Ziheng Zhao, Chunliang Qi, Yu Zhang, Wei Wang

**Affiliations:** 1 NHC Key Laboratory of Medical Immunology, Medicine Innovation Center for Fundamental Research on Major Immunology-Related Diseases, Department of Immunology, School of Basic Medical Sciences, Peking University, 38 Xueyuan Road, Haidian District, Beijing 100191, China; wuzhaofei@pku.edu.cn (Z.W.); liuyuwei0416@163.com (Y.L.); 1810305326@pku.edu.cn (J.W.); zhaoziheng@pku.edu.cn (Z.Z.); 1810305202@pku.edu.cn (C.Q.); 2 NHC Key Laboratory of Medical Immunology, Department of Microbiology and Infectious Disease Center, School of Basic Medical Sciences, Peking University, 38 Xueyuan Road, Haidian District, Beijing 100191, China; xianwei@pku.edu.cn

**Keywords:** lactate, treg, MCT4, lactylation, tumor immunity

## Abstract

High lactate concentration is a hallmark of the tumor microenvironment (TME). Regulatory T cells (Tregs) exhibit unique metabolic adaptability to this lactate-rich environment, yet the underlying mechanisms remain incompletely understood. Here, we demonstrate that the monocarboxylate transporter MCT4 is upregulated in tumor-infiltrating Tregs and mediates direct lactate uptake. Using Treg-specific conditional knockout (cKO) mice, we show that MCT4 deficiency does not affect basal Treg development but abrogates lactate-induced Foxp3 stabilization and impairs Treg suppressive function. Mechanistically, MCT4-mediated lactate uptake promotes the lactylation of Foxp3 at lysine 277 (K277), which competitively inhibits its ubiquitination, thereby enhancing Foxp3 protein stability and nuclear localization. Nuclear Foxp3 subsequently interacts with IRF3 to promote IL-10 transcription and secretion. In the B16 melanoma model, MCT4-deficient Tregs display compromised stability and reduced tumor infiltration, leading to enhanced CD8^+^ T cell effector function and attenuated tumor growth. Collectively, our findings reveal that MCT4-mediated lactate uptake sustains Treg stability and function through Foxp3 lactylation, identifying MCT4 as a potential therapeutic target for modulating Treg activity in cancer.

## 1. Introduction

Tregs, a subset of CD4^+^ T cells characterized by the high expression of CD25 and the transcription factor Foxp3, play critical roles in maintaining immune homeostasis while also contributing to inflammatory diseases and tumor immune evasion [1,2,3,4,5]. Clinical studies have shown that Tregs are significantly enriched in various solid tumors, and their infiltration levels correlate with poor patient prognosis [6,7,8]. Tregs employ multiple mechanisms to suppress immune responses. In a contact-dependent manner, Tregs highly express immune checkpoint molecules including CTLA-4, PD-1 and PD-L1 [9,10,11], which directly inhibit effector T cell activation. In a contact-independent manner, Tregs consume IL-2 in the microenvironment through high CD25 expression, thereby inhibiting effector T cell proliferation and activation [1,12,13]; Tregs also secrete anti-inflammatory cytokines such as IL-10, IL-35 and TGF-β to exert paracrine immunosuppressive effects [14,15]. IL-10 is critically involved in Treg function in infectious and autoimmune diseases [16,17]. Mechanistically, IL-10 indirectly suppresses Th17 and Th1 differentiation by inhibiting TNF-α and IL-1β secretion from monocytes and macrophages [18], suppressing dendritic cell antigen presentation [19], and reducing IL-6 and IL-12 production [20,21]. In tumor immunity, IL-10 and IL-35 secreted by Tregs promote CD8^+^ T cell exhaustion [22]. IL-10 neutralizing antibodies block Treg-mediated immunosuppression in chronic lymphocytic leukemia and melanoma [22,23]. These findings suggest that IL-10 is a key effector molecule mediating Treg immunosuppressive function, and targeting the IL-10 signaling pathway may represent a potential strategy for cancer immunotherapy [24,25,26].

TME is defined by hypoxia, low glucose, and high lactate. It has been reported that the lactate concentration in human serum is approximately 1 mM, whereas in tumor tissue, due to the Warburg effect, lactate levels can rise to 10–30 mM [27,28,29]. High levels of lactate broadly influence the immune response within the TME: they inhibit dendritic cell differentiation and antigen presentation, promote macrophage polarization toward the M2 phenotype, suppress the immune surveillance capacity of natural killer cells and T cells, and inhibit the function and reduce the survival of group 2 innate lymphoid cells (ILC2s) [30,31,32,33,34]. Compared with conventional T cells (Tconv), Tregs exhibit a distinctive metabolic adaptability that facilitates their survival in low-glucose, high-lactate conditions. Mechanistically, Foxp3 suppresses glycolytic activity in Tregs, rendering their energy metabolism less dependent on glycolysis while enhancing lactate utilization to maintain NAD^+^/NADH homeostasis, thereby enabling adaptation to the metabolic stress of TME [35]. Further studies have identified a lactate-affinity subset of tumor-infiltrating Tregs with high MCT1 expression that displays higher lactate dehydrogenase activity and stronger immunosuppressive function compared with the glucose-affinity subset with low MCT1 expression [35,36]. Lactate is primarily transported across the membrane by the monocarboxylate transporter (MCT) family members MCT1 and MCT4 [37,38]. MCT1 is induced by c-Myc and widely expressed in various cell types, whereas MCT4 is induced by HIF-1α and is highly expressed in glycolytically active tissues such as white muscle fibers and cancer cells [39,40,41]. Most solid tumors rely on glycolysis for energy production and export large amounts of lactate via MCT4 to maintain intracellular pH homeostasis [28]. High expression of MCT1 and MCT4 in various tumors, including melanoma [42], glioblastoma [43], and non-small cell lung cancer [44], correlates with poor prognosis.

Beyond serving as a metabolic substrate, lactate modulates Treg function through multiple pathways: it promotes TGF-β receptor membrane localization to enhance Foxp3 induction [45]; generates phosphoenolpyruvate that activates NFAT1 via Ca^2+^ influx, leading to PD-1 upregulation [46]; and facilitates USP39 mRNA splicing to regulate CTLA-4 membrane expression [47]. While the critical role of lactate in maintaining Treg stability and enhancing immunosuppressive function is established, the specific impact of the HIF-1α-induced MCT4 axis remains elusive. Given that TME is characterized by hypoxia, where tumor-infiltrating Tregs rely on HIF-1α for differentiation and stability [48,49,50,51], we hypothesize that MCT4 may serve as a key mediator of lactate dynamics, thereby dictating the functional fate of tumor-associated Tregs.

Lysine lactylation, a novel post-translational modification first reported in 2019, uses lactate as a substrate to modify histone lysine residues and participates in gene expression regulation [52]. Subsequent studies have identified the writer p300 [52] and the erasers HDAC1-3 [53] and SIRT1-3 [54,55] for this modification. Recent findings have identified AARS1 and AARS2 as lactate sensors and transferases that mediate lactylation of non-histone proteins such as p53, YAP, and mitochondrial proteins [56,57,58]. Mass spectrometry data indicate that lactylation is widespread among extranuclear proteins and participates in various physiological and pathological processes by regulating protein stability and function [59]. However, direct links between lactylation and Treg functional regulation remain scarce. Gu et al. reported that moesin lactylation promotes TGF-β receptor membrane localization, enhancing Foxp3 induction via TGF-β signaling [45], while Wang et al. demonstrated that in Foxp3^+^ natural killer T cells, lactate promotes H3K18 lactylation at the *Foxp3* gene promoter, upregulating Foxp3 expression [60]. As the master transcription factor that maintains Treg immunosuppressive function, Foxp3 is an ideal candidate for investigating how post-translational modifications, particularly lactylation, regulate Treg function in the TME. Elucidating these mechanisms may uncover new targets for Treg-directed cancer therapy.

To this end, we propose the central hypothesis that MCT4-mediated lactate transport in Tregs elevates intracellular lactate levels, which in turn induces Foxp3 lactylation and enhances Treg immunosuppressive function. To test this hypothesis, we investigated the effects of lactate on Treg function using high-concentration sodium lactate (NaLa) to mimic the lactate-rich TME, defined the role of MCT4 in lactate transport using Treg-specific MCT4 cKO mice, identified key lactylation sites on Foxp3 and characterized their impact on Foxp3 stability and transcriptional activity, dissected the molecular mechanisms by which lactylated Foxp3 regulates downstream effectors such as IL-10, and validated the functional significance of MCT4-mediated lactate uptake in Treg stability and antitumor immune responses using multiple murine tumor models. Collectively, these approaches delineate the role of the lactate–MCT4–Foxp3 lactylation axis in Treg functional regulation within the TME.

## 2. Results

### 2.1. MCT4 Mediates Lactate Uptake in Tumor-Infiltrated Tregs

**Figure 1:**
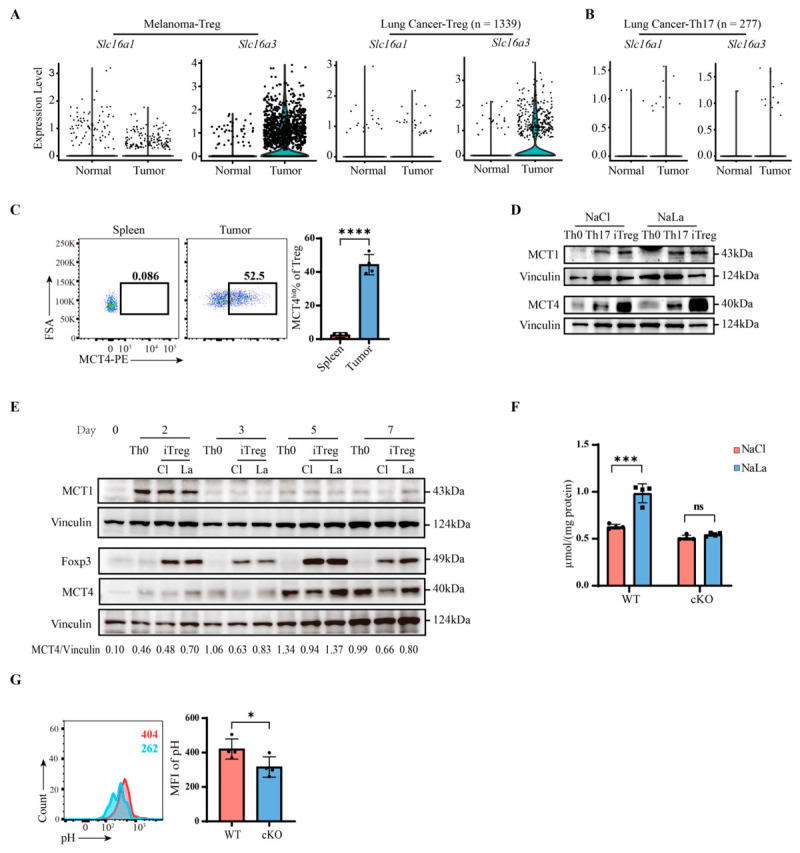
MCT4 mediates lactate uptake in tumor-infiltrated Tregs. (**A**,**B**) Analysis of Slc16a1 and Slc16a3 mRNA expression levels in Treg and Th17 subsets infiltrating tumor and normal tissues from published single-cell RNA-Seq datasets GSE126184 (mouse melanoma) and GSE152022 (mouse lung cancer). Normal, adjacent normal tissue; tumor, tumor tissue. “*n*” represents the number of Treg and Th17 cells used for analysis. (**C**) Flow cytometry analysis of MCT4 expression levels in splenic and tumor-infiltrating Tregs 14 days after B16 melanoma inoculation, *n* = 4. (**D**) Western blotting analysis of MCT1 and MCT4 expression in naive CD4^+^ T cells cultured under Th0, Th17, or Treg polarization conditions for 3 days with 10 mM NaCl or NaLa. (**E**) Naive CD4^+^ T cells were cultured under Th0 or Treg polarization conditions from day 0–3, then rested without TCR or TGF-β stimulation from day 3–5, and restimulated with TCR from day 5–7. “Day” indicates time points at which the cells were harvested; “Th0” and “iTreg” indicate polarization conditions during the first 3 days, with identical treatments from days 3–7; “Cl” and “La” indicate 10 mM NaCl and NaLa, respectively. (**F**) ELISA measurement of intracellular lactate levels in cKO Tregs from stability experiments, normalized to total cellular protein. *n* = 4. (**G**) Flow cytometry analysis of tumor-infiltrating WT (YFP^−^) and cKO (YFP^+^) Tregs from *Slc16a3^flox/flox^Foxp3^YFP-cre/+^* mice bearing B16 melanomas stained with a pH probe. Numbers indicate mean the fluorescence intensity of the pH probe; right panel shows the quantification of pH probe MFI. *n* = 4. Statistical analysis was performed using the Student’s *t*-test. * *p* < 0.05, *** *p* < 0.001, **** *p* < 0.0001.

To investigate the molecular mechanism underlying lactate uptake in tumor-infiltrating Tregs, we first analyzed published single-cell RNA-Seq datasets [22,61] to compare the expression patterns of the two main lactate transporters on T cells, MCT1 (encoded by *Slc16a1*), and MCT4 (encoded by *Slc16a3*). The results showed that *Slc16a3* expression was significantly upregulated in melanoma- and lung cancer-infiltrating Tregs compared with Tregs from normal tissues, whereas *Slc16a1* expression showed no significant difference (Figure 1A). In contrast, Th17 cells exhibited low expression of both genes in both normal and tumor tissues (Figure 1B and Figure S1A). Later, using the B16 melanoma mouse model, we confirmed that MCT4 protein levels were elevated in tumor-infiltrating Tregs, since there was barely any expression in splenic Tregs (Figure 1C).

We next examined MCT1 and MCT4 expression in vitro. Consistent with the single-cell transcriptomic data, naïve CD4^+^ T cells displayed distinct MCT1 and MCT4 expression patterns under different polarization conditions. Th0 cells expressed low levels of both transporters; Th17 cells showed a moderate upregulation of both, whereas Treg cells exhibited a striking and selective upregulation of MCT4, which was further enhanced by exogenous sodium lactate (NaLa) treatment, with MCT1 expression remaining unchanged (Figure 1D). Moreover, kinetic analysis using a sequential differentiation and restimulation model in vitro revealed distinct temporal expression patterns of MCT1 and MCT4. Naïve CD4^+^ T cells were polarized toward induced Tregs (iTregs) for 3 days, followed by 2 days of resting to allow for expansion, and then purified the iTregs were restimulated for an additional 2 days. Under this experimental scheme, MCT1 expression on iTregs peaked on day 2 and then declined rapidly (“fast up, fast down”), a similar pattern as that reported in C2C12 myoblasts [62]; in contrast, MCT4 expression emerged later, peaked around day 5, and remained elevated through day 7 (Figure 1E). This sustained MCT4 expression correlated with increased Foxp3 protein levels after restimulation, suggesting that MCT4 did not primarily intervene in early Treg differentiation but rather supported Foxp3 stability in already committed Tregs under chronic high-lactate conditions.

To explore the function of MCT4 in Tregs, we generated Treg-specific *Slc16a3* conditional knockout (cKO) mice (*Slc16a3^flox/flox^Foxp3^YFP-cre^*). Efficient ablation of MCT4 in cKO Tregs was confirmed (Figure S1B,C), and no significant differences in T cell subset development were observed in the thymus or spleen between WT and cKO mice (Figure S1D,E), indicating that MCT4 deficiency does not affect T cell development or maturation.

Given that MCT4 has been characterized as a lactate exporter in tumor cells [63], we initially expected that MCT deficiency would result in intracellular lactate accumulation upon exogenous NaLa treatment. Surprisingly, however, the MCT4 cKO Tregs exhibited no significant chance in intracellular lactate levels, whereas WT Tregs showed a marked increase in lactate uptake and elevated intracellular lactate content (Figure 1F). These data indicate that MCT4 in Tregs may be not responsible for lactate export but rather for lactate uptake in Tregs. To further assess this possibility and investigate the functional role of MCT4 in Tregs in vivo, we utilized a pH probe as an indirect indicator of intracellular lactate levels. Using *Slc16a3^flox/flox^Foxp3^YFP-cre/+^* female mice bearing B16 melanomas, in which approximately half of the Tregs are cKO (YFP^+^) and the other half are WT (YFP^−^), we directly compared the two populations within the same TME. cKO Tregs did exhibit lower pH probe mean fluorescence intensity (MFI) than WT Tregs (Figure 1G). Collectively, these results demonstrate that MCT4 was highly expressed in tumor-infiltrating Tregs in correlation with enhanced Foxp3 expression, and that, contrary to its canonical role as a lactate exporter in other cell types, MCT4 in Tregs may mediate lactate uptake but not efflux in TME.

### 2.2. Uptake of Lactate by MCT4 Serves to Enhance Foxp3 Stability via K277 Lactylation

**Figure 2:**
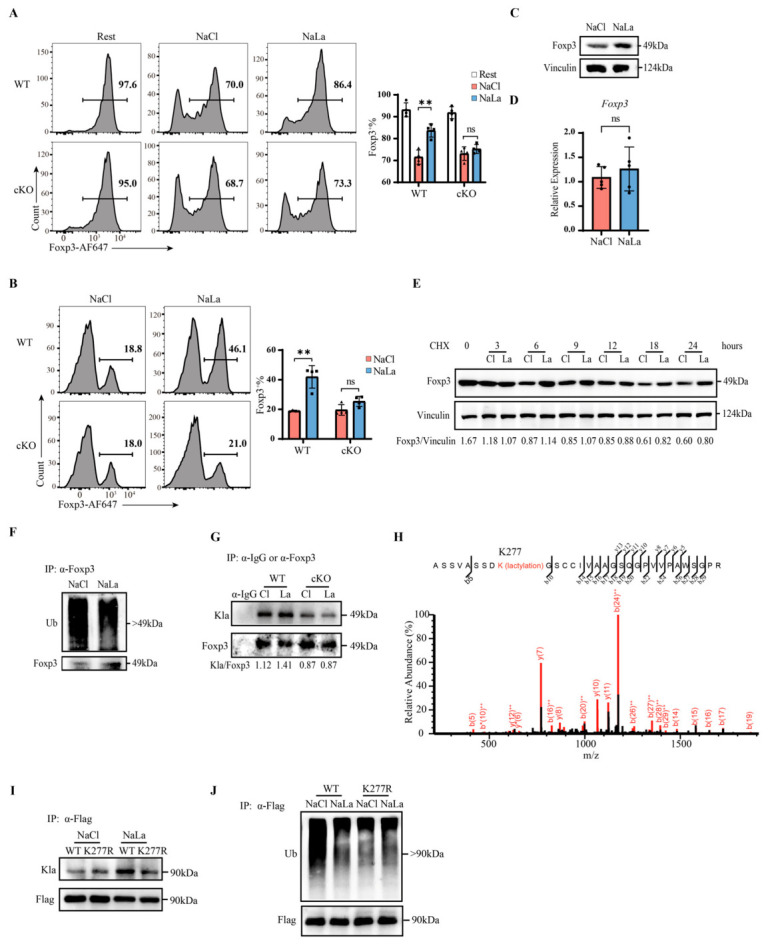
Uptake of lactate by MCT4 serves to enhance Foxp3 stability via K277 lactylation. (**A**) In vitro stability assay of cKO Tregs. Flow cytometry analysis of Foxp3 expression. Rest indicates no TCR restimulation during the stability assay. *n* = 4. (**B**) Sorted Tregs were injected intravenously into *Rag1^−/−^* mice. After 14 days, splenocytes were harvested and Foxp3 expression was analyzed by flow cytometry within CD45.2^+^CD4^+^ cells. *n* = 4. (**C**) Western blotting analysis of Foxp3 in Treg stability assay samples treated with 10 mM NaCl or NaLa. (**D**) Foxp3 mRNA expression in Tregs obtained after induction, expansion, and sorting. A total of 10 Mm NaCl or NaLa was added during the expansion phase. *n* = 5. (**E**) Western blotting analysis of Tregs treated with 5 μM CHX and 10 mM NaCl or NaLa for indicated times. (**F**) Immunoprecipitation-western blotting analysis of Tregs using anti-Foxp3 antibody after stimulation with 10 mM NaCl or NaLa for 48 h. (**G**) Immunoprecipitation-Western blotting analysis of Tregs using anti-Foxp3 antibody. Tregs were induced from naive T cells and treated with 10 mM NaCl (Cl) or NaLa (La). Kla, lactylation. (**H**) Mass spectrometry analysis of FOXP3 K277 lactylation. “*” indicates a neutral loss during fragmentation of the precursor ion. (**I**,**J**) HEK293T cells overexpressing WT or K277R FOXP3-GFP-Flag were treated with 20 mM NaLa for 48 h and subjected to immunoprecipitation-Western blotting analysis using the anti-Flag antibody. WT, wild-type FOXP3; K277R, FOXP3 K277R mutant. Kla, lactylation; Ub, ubiquitination. Statistical analysis was performed using the Student’s *t*-test. ** *p* < 0.01, ns, *p* > 0.05.

Foxp3 expression is susceptible to microenvironmental influences. We therefore investigated the role of MCT4 in maintaining Foxp3 stability. In vitro stability assays showed that under control (NaCl) conditions, Foxp3 expression levels in cKO Tregs were comparable to those in WT Tregs; however, NaLa treatment significantly inhibited the degradation of Foxp3 induced by re-stimulation in WT Tregs but failed to sustain Foxp3 expression in cKO Tregs (Figure 2A). In vivo adoptive transfer experiments further confirmed these findings: WT and cKO Tregs were induced, expanded with or without NaLa treatment, sorted, and injected intravenously into *Rag1^−/−^* mice. NaLa pretreatment obviously maintained Foxp3 expression in WT Tregs but had no protective effect on cKO Tregs (Figure 2B). These results indicate that MCT4 deficiency did not affect basal Treg stability but abrogated the response to extracellular lactate.

To determine whether NaLa affected Foxp3 protein stability or its transcriptional efficiency, we examined both its protein and mRNA levels. As shown in Figure 2C,D, NaLa treatment only influenced the protein levels but did not affect mRNA. Although lactate has been reported to regulate Foxp3 expression via H3K18 lactylation under Th17-polarizing conditions [64], we observed no significant change in H3K18 lactylation levels in Tregs under our experimental conditions (Figure S2B). This suggests that the effect of NaLa, mediated by MCT4, on Treg stability occurs primarily at the post-translational level. Later, cycloheximide (CHX) experiments also showed that NaLa treatment significantly extended the half-life of Foxp3 protein (Figure 2E and Figure S2C). Further analysis revealed that the NaLa effect was significantly on Foxp3 ubiquitination (Figure 2F). To figure out how lactate affects the post-translational level of Foxp3, we overexpressed FOXP3-Flag in HEK293T cells. We found that NaLa treatment could decrease ubiquitination and increased the lactylation of FOXP3, but did not affect the phosphorylation or acetylation levels (Figure S2D–G). Immunoprecipitation experiments in primary Tregs confirmed that NaLa treatment significantly enhanced Foxp3 lactylation in WT Tregs, an effect that was completely abolished in cKO Tregs (Figure 2G), indicating that MCT4-mediated lactate uptake promotes Foxp3 lactylation.

Lactylation is a recently identified post-translational modification [52] that includes both histone lactylation, which regulates target gene transcription and the lactylation of mature proteins, modulating their function. In this context, since maintaining Foxp3 protein expression is of greater significance in already differentiated Treg cells and its transcription remains unchanged, we consider it more likely that Foxp3 undergoes lactylation at the protein level. To identify the specific lactylation sites on Foxp3, we performed immunoprecipitation and conducted mass spectrometry analysis on FOXP3 overexpressed in HEK293T cells. Five lactylated lysine residues were identified: K8, K200, K206, K216, and K277 (Figure 2H and Figure S2E), all of which are conserved between human and mouse. Among these, K277 showed the highest lactylation abundance, accounting for 41.3% of the total signals. To validate the functional significance of K277, we generated a K277R point mutation construct. Compared with WT FOXP3, the K277R mutant exhibited no alteration in lactylation upon NaLa treatment (Figure 2I) and maintained lower ubiquitination levels (Figure 2J). In contrast, the K393R mutation did not affect FOXP3 lactylation (Figure S2F). In summary, these results demonstrate that NaLa treatment promoted Foxp3 lactylation at K277, which competed with ubiquitination at the same residue, thereby enhancing Foxp3 protein stability.

### 2.3. Lactylated Foxp3 Promotes IL-10 Secretion, Enhancing Treg Suppressive Function

**Figure 3:**
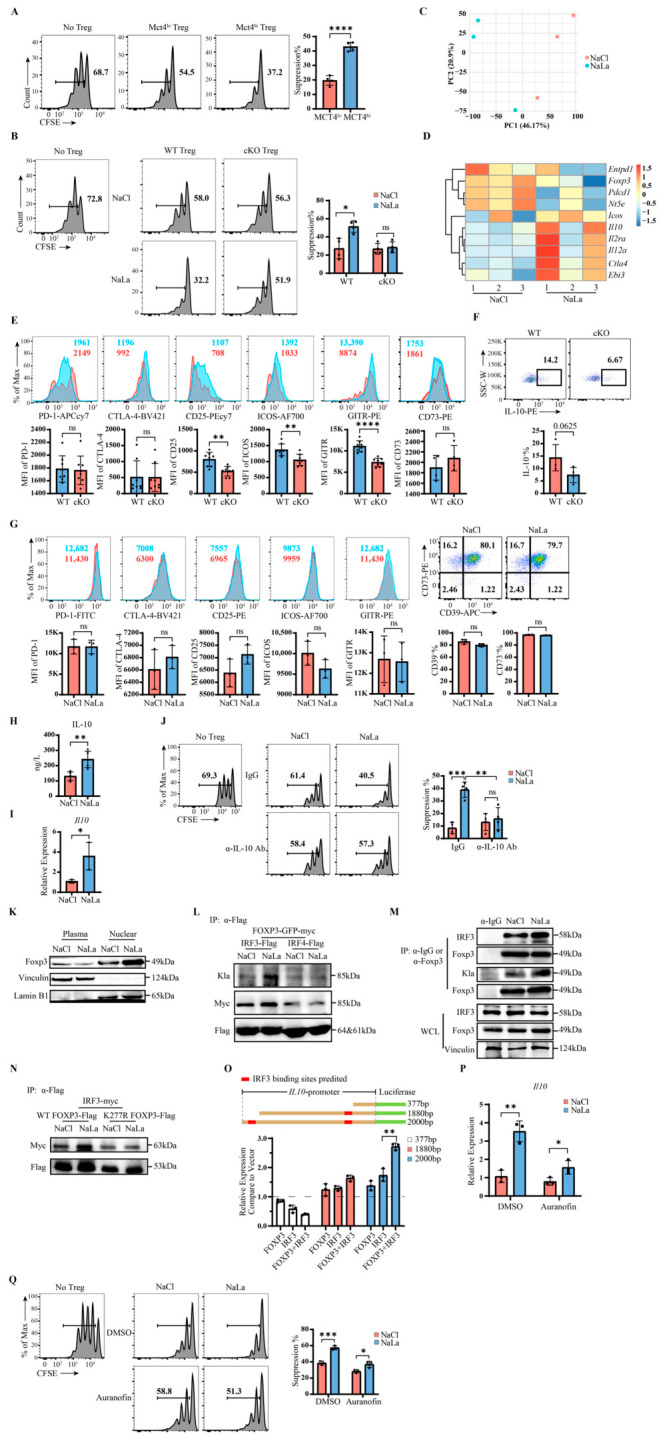
Lactylated Foxp3 promotes IL-10 secretion, enhancing Treg suppressive function. (**A**) In vitro Treg suppression assay using MCT4^lo^ and MCT4^hi^ Tregs sorted from B16 melanomas 10–14 days after inoculation. Left panel shows CFSE proliferation peaks of co-cultured naive T cells; right panel shows quantification of naive T cell proliferation inhibition. *n* = 4. (**B**) In vitro Treg suppression assay using WT and cKO Tregs induced from naive CD4^+^ T cells and pretreated with 10 mM NaCl or NaLa. Left panel shows CFSE proliferation peaks of co-cultured naive T cells; right panel shows quantification of naive T cell proliferation inhibition. *n* = 4. (**C**,**D**) RNA-Seq analysis of sorted Treg from the stability experiments treated with 10 mM NaCl or NaLa for 48 h. PCA analysis (**C**) and expression of immunosuppressive molecules (**D**). (**E**) Flow cytometry analysis of PD-1, CTLA-4, CD25, ICOS, GITR, and CD73 in tumor-infiltrating Tregs from *Slc16a3^flox/flox^Foxp3^YFP-cre/EGFP^* mice bearing B16 melanomas. “K” denotes thousand (e.g., 15K = 15,000). *n* = 8 for all except CD73 (*n* = 4). (**F**) Flow cytometry analysis of IL-10 in tumor-infiltrating Tregs from *Slc16a3^+/+^Foxp3^YFP-cre^* WT and *Slc16a3^flox/flox^Foxp3^YFP-cre^*cKO mice bearing B16 melanomas. *n* = 4, *p* = 0.0625. (**G**) Flow cytometry analysis of non-secretory immunosuppressive molecules in Treg stability assay samples. “K” denotes thousand (e.g., 14K = 14,000). *n* = 3. (**H**) ELISA measurement of IL-10 in Treg culture supernatants from stability experiments. *n* = 5. (**I**) *Il10* mRNA expression in Tregs from stability experiments. *n* = 3. (**J**) Treg suppression assay using Tregs pretreated with 10 mM NaCl or NaLa. IgG or anti–mouse IL-10 neutralizing antibody (500 ng/mL) was added during the co-culture phase. Left panel shows the CFSE proliferation peaks of co-cultured naive T cells; right panel shows quantification of naive T cell proliferation inhibition. *n* = 4. (**K**) Western blotting analysis of Foxp3 in nuclear and cytoplasmic fractions from Treg stability assay samples. (**L**) HEK293T cells co-transfected with FOXP3-GFP-myc and IRF3-Flag or IRF4-Flag were treated with 20 mM NaCl or NaLa for 48 h and subjected to immunoprecipitation-Western blotting analysis. Kla, lactylation. (**M**) Immunoprecipitation-Western blotting analysis of expanded Tregs using anti-Foxp3 antibody after treatment with 10 mM NaCl or NaLa for 48 h. Kla, lactylation. (**N**) HEK293T cells co-transfected with WT FOXP3-Flag or K277R FOXP3-Flag and IRF3-Myc were treated with 20 mM NaCl or NaLa for 48 h and subjected to immunoprecipitation-Western blotting analysis. (**O**) Dual luciferase reporter assay in HEK293T cells using *IL10* promoter constructs containing different numbers (0–2) of predicted IRF3 binding sites. Foxp3 + IRF3 indicates co-transfection of Foxp3 and IRF3. *n* = 3. (**P**) *Il10* mRNA expression in Tregs treated with 100 nM IRF3 inhibitor auranofin from the stability experiments, 0.1% DMSO was used as the control. *n* = 3. (**Q**) Treg suppression assay using sorted iTregs pretreated with 10 mM NaCl or NaLa and 100 nM IRF3 inhibitor auranofin or 0.1% DMSO for 48 h. Left panel shows CFSE proliferation peaks of co-cultured naive T cells; right panel shows quantification of naive T cell proliferation inhibition. *n* = 3. Statistical analysis was performed using the Student’s *t*-test. * *p* < 0.05, ** *p* < 0.01, *** *p* < 0.001, **** *p* < 0.0001, ns, *p* > 0.05.

To examine whether lactate uptake via MCT4 also affected the Treg immunosuppressive capacity, we established a B16 mouse model. Tumor-infiltrating Tregs were sorted into MCT4^hi^ and MCT4^lo^ populations based on the MCT4 expression levels and subjected to in vitro suppression assays. Tregs from the MCT4^hi^ group exhibited stronger suppressive capacity than those from the MCT4^lo^ group (Figure 3A). Using cKO mice, naïve CD4^+^ T cells were induced into Tregs, pretreated with NaCl or NaLa, and then sorted out for in vitro suppression assays. As we seen in Figure 3B, NaLa pretreatment enhanced the suppressive function of WT Tregs (51.44% vs. 27.23%), but had no effect on cKO Tregs (28.61% vs. 26.89%), consistent with the understanding that MCT4 deficiency abrogates Treg responsiveness to NaLa.

RNA-Seq analysis was performed on NaLa-treated Tregs to explore its downstream effect (Figure 3C,D). The results showed that NaLa treatment downregulated the expression of *Pdcd1*, *Entpd1*, and *Nt5e* while upregulating *Icos*, *Il10*, *Il2ra*, *Il12a*, *Ctla4*, and *Ebi3*. Using *Slc16a3^flox/flox^Foxp3^YFP-cre/EGFP^* mice, we screened immunosuppressive molecules in tumor-infiltrating Tregs and found that WT Tregs exhibited a higher expression of CD25, GITR, and ICOS compared with cKO Tregs, whereas PD-1, CTLA-4, and CD73 showed no significant differences (Figure 3E); IL-10 secretion was also higher in WT Tregs (Figure 3F). In vitro stability assays revealed no significant differences in PD-1, CTLA-4, ICOS, GITR, CD39, or CD73 expression, except for an upward trend in CD25 (Figure 3G). Cytokine analysis showed that NaLa treatment significantly increased IL-10 secretion but did not affect the IL-35 levels (Figure 3H and Figure S3A). mRNA expression analysis revealed no significant changes in *Pdcd1*, *Il2ra*, *Entpd1*, *Nt5e*, *Il12a*, or *Ebi3* (Figure S3B), whereas *Il10* expression was significantly upregulated (Figure 3I). These results indicate that NaLa treatment specifically promotes IL-10 expression and secretion in Tregs, and that MCT4-deficient Tregs exhibit attenuated lactate responsiveness.

To determine whether IL-10 mediates NaLa-induced enhancement of Treg suppressive function, we performed suppression assays in the presence of the anti-IL-10 neutralizing antibody. In the IgG control group, NaLa pretreatment significantly enhanced Treg suppressive capacity; this effect was completely abrogated in the IL-10 neutralizing antibody group (Figure 3J), establishing IL-10 as a critical downstream effector of NaLa-mediated Treg functional modulation.

Subcellular fractionation experiments showed that NaLa treatment increased the nuclear distribution of Foxp3 (Figure 3K). IL-10 expression in Tregs is known to be regulated by Blimp1 and IRF4, with multiple IRF4 binding sites present in the *Il10* promoter [65]; IRF3, another member of the IRF family, has also been reported to directly bind the *Il10* promoter in regulatory B cells [66]. To explore whether Foxp3 interacts with IRF family members to regulate IL-10 transcription, we coexpressed FOXP3-Myc with IRF3-Flag or IRF4-Flag in HEK293T cells. Co-immunoprecipitation experiments revealed that NaLa treatment significantly enhanced the interaction between FOXP3 and IRF3, whereas the interaction with IRF4 remained unchanged (Figure 3L). This finding was further validated in primary Tregs, where Foxp3 exhibited higher lactylation levels upon interaction with IRF3, suggesting that lactylated Foxp3 possesses an enhanced competitive ability to bind IRF3 (Figure 3M). Notably, this enhanced interaction is dependent on Foxp3 lactylation, as the K277R mutation abolished the NaLa-induced increase in the FOXP3–IRF3 binding, demonstrating that lactate acted through Foxp3 lactylation rather than directly on IRF3 (Figure 3N). JASPAR database analysis predicted two IRF3 binding sites within the *IL10* promoter region, 2000 bp upstream of the transcription start site. Luciferase reporter assays using *IL10* promoter constructs containing different numbers of predicted binding sites showed that co-transfection of FOXP3 and IRF3 significantly enhanced promoter activity in a manner dependent on the presence of these binding sites (Figure 3O). Anna Glanz et al. reported that auranofin is a novel inhibitor of IRF3 functions [67]. Consistently, the results from the mRNA expression analysis and Treg suppression assay revealed that auranofin treatment attenuated the NaLa-induced increase in *Il10* expression (Figure 3P) and therefore Tregs’ suppressive capacity (Figure 3Q), suggesting that the effect of NaLa on IL-10 and Treg cells requires IRF3. Collectively, these results demonstrate that lactylated Foxp3 interacts with IRF3 to promote *Il10* transcription.

### 2.4. Lactate Uptake by MCT4 Is Essential for Maintaining Tumor-Infiltrating Treg Stability

**Figure 4:**
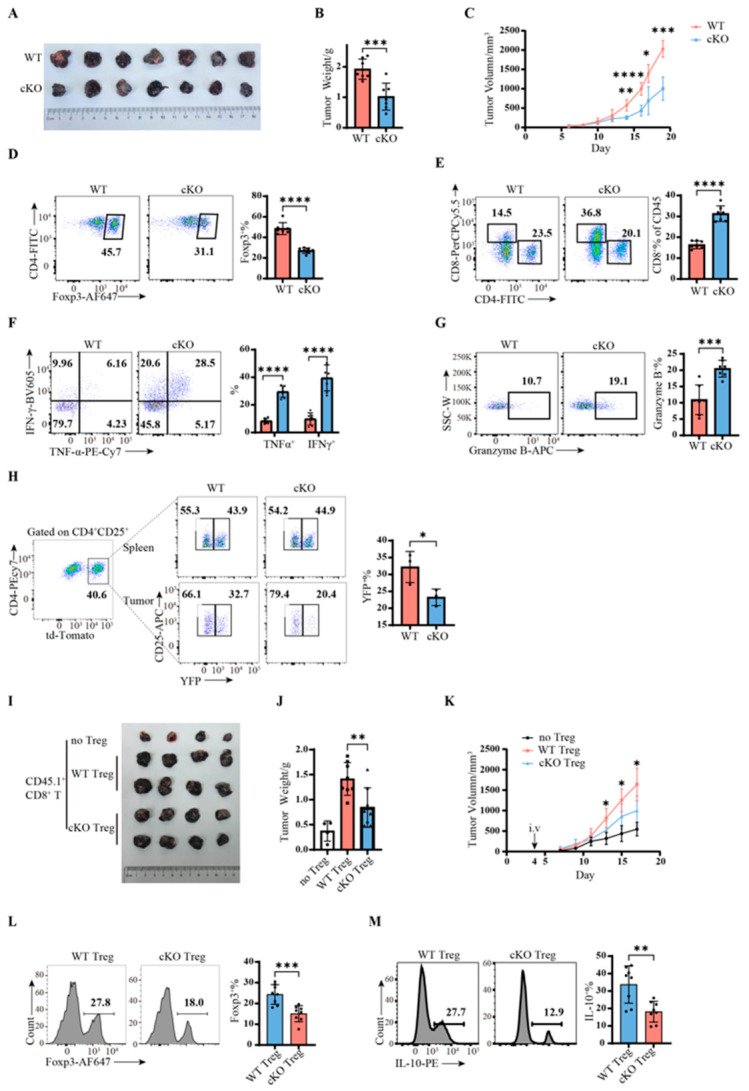
Lactate uptake by MCT4 is essential for maintaining tumor-infiltrating Treg stability. (**A**–**G**) B16 melanoma model in *Slc16a3^flox/flox^Foxp3^YFP-cre^* mice. *n* = 7. (**A**) Tumor size at harvest on day 20. (**B**) Tumor weight at harvest on day 20. (**C**) Tumor growth curve. (**D**) Proportion of tumor-infiltrating Tregs among CD4^+^ T cells. (**E**) Proportion of tumor-infiltrating CD8^+^ and CD4^+^ T cells among CD45^+^ cells. Quantification shown only for CD8^+^ T cells on the right. (**F**,**G**) Cytokine production by tumor-infiltrating CD8^+^ T cells: IFN-γ, TNF-α, and granzyme B. (**H**) Proportion of YFP^+^ and YFP^−^ cells among the CD4^+^CD25^+^tdTomato^+^ tumor-infiltrating Tregs from *Slc16a3^flox/flox^Foxp3^YFP-cre^Rosa26^tdTomato/+^* cKO mice and *Slc16a3^+/+^Foxp3^YFP-cre^Rosa26^tdTomato/+^* WT mice. *n*= 3. (**I**–**M**) B16 melanoma model in *Rag1*^−/−^ mice with adoptive transfer of CD8^+^ T and/or Treg cells. *n* = 4 or 8. (**I**) Tumor size at harvest on day 18. (**J**) Tumor weight at harvest on day 18. (**K**) Tumor growth curve. * *p* < 0.05 for cKO Treg vs. WT Treg. (**L**) Proportion of Foxp3^+^ cells among CD45.2^+^CD4^+^ cells. (**M**) Proportion of IL-10^+^ cells among Tregs. Statistical analysis was performed using the Student’s *t*-test or two-way ANOVA. * *p* < 0.05, ** *p* < 0.01, *** *p* < 0.001, **** *p* < 0.0001.

Our in vitro findings indicated that MCT4 plays a critical role in maintaining Treg function under high-lactate conditions. To validate this in vivo, we established a sub.q B16 melanoma model, which exhibits high lactate levels and strong immunogenicity. cKO mice showed significantly slower tumor growth compared with WT mice (Figure 4C), with reduced tumor volume and weight (Figure 4A,B). Analysis of the tumor-infiltrating immune cell composition revealed a significant reduction in Treg infiltration in cKO mice (Figure 4D), indicating impaired Treg immunosuppressive function due to MCT4 deficiency in the high-lactate TME. Concomitantly, cKO mice exhibited increased infiltration of CD8^+^ T cells and enhanced production of IFN-γ, TNF-α, and granzyme B (Figure 4E–G), indicative of enhanced antitumor immune responses. Notably, no significant differences in Treg or CD8^+^ T cell proportions or cytokine production were observed in draining lymph nodes between WT and cKO mice, where there was barely MCT4 expression on Tregs (Figure S4A–D). These results align with our in vitro observations, demonstrating that MCT4 deficiency affects Treg function in the lactate-rich TME, underscoring the importance of MCT4 expression in Treg adaptation to high-lactate environments.

To exclude potential confounding effects from differential tumor growth rates between WT and cKO mice, we repeated the melanoma experiment using *Slc16a3^flox/flox^Foxp3^YFP-cre/+^* female mice, in which both WT (YFP^−^) and cKO (YFP^+^) Tregs coexist within the same TME. Compared with the spleen, WT Tregs showed increased infiltration into tumors, whereas cKO Tregs showed decreased infiltration (Figure S4E), confirming that MCT4 deficiency directly impairs Treg infiltration capacity. To further refine this analysis, we crossed cKO mice with *Foxp3*-EGFP reporter mice to generate *Slc16a3^flox/flox^Foxp3^YFP-cre/EGFP^* mice, enabling a clear discrimination between WT (EGFP^+^) and cKO (YFP^+^) Tregs based on fluorescence intensity in the FITC channel. Consistent results were obtained (Figure S4F), and pH probe staining confirmed that cKO Tregs exhibited higher intracellular pH and lower lactate levels (Figure S4G), consistent with Figure 1G.

We systematically investigated the potential mechanisms underlying reduced Treg infiltration in cKO mice, including increased apoptosis, decreased proliferation, impaired chemotaxis, compromised stability, and altered induction. No significant differences were observed in Annexin V/7-AAD staining (Figure S4H), chemokine receptor expression (CCR4, CCR5, CCR6, CCR8, CCR10) (Figure S4J), or Ki67 proliferation (Figure S4I) between the WT and cKO Tregs. To assess stability, we crossed *Slc16a3^flox/flox^Foxp3^YFP-cre^* mice with *Rosa26^tdTomat^^o^* reporter mice, enabling lineage tracing of Tregs. In this system, cells that have ever expressed Foxp3 irreversibly express tdTomato, allowing for the identification of cells that have lost Foxp3 expression (unstable) versus those that never expressed Foxp3. Tumor-infiltrating cKO Tregs exhibited a significantly higher proportion of unstable cells compared with WT Tregs (Figure 4H), indicating that exacerbated instability in the high-lactate environment is a key mechanism underlying the reduced infiltration of cKO Tregs.

Having established that compromised stability is the key mechanism for reduced infiltration of MCT4-deficient Tregs, we next evaluated whether this instability alone is sufficient to slow tumor growth. We performed adoptive transfer experiments in *Rag1^−/−^* mice, which lack endogenous mature T and B lymphocytes, thereby excluding potential confounding from Treg induction. Sorted WT or cKO Tregs (purity ~97%; Figure S4K) were adoptively transferred into *Rag1^−/−^* mice bearing B16 tumors. Mice that did not receive Tregs exhibited the slowest tumor growth. Compared with mice receiving WT Tregs, those receiving cKO Tregs showed significantly slower tumor growth (Figure 4K), with reduced tumor volume and weight (Figure 4I,J). Analysis of tumor-infiltrating CD4^+^ T cells revealed that cKO Tregs had a significantly lower proportion of Foxp3^+^ cells (Figure 4L) and IL-10^+^ cells (Figure 4M) compared with WT Tregs. These results provide definitive evidence that compromised stability of MCT4-deficient Tregs is a key biological cause of reduced tumor growth.

## 3. Discussion

In this study, we found that under high-lactate conditions, Tregs upregulated MCT4 expression and took up lactate. This lactate uptake promoted Foxp3 lactylation at K277, which rendered Foxp3 resistant to ubiquitin-mediated degradation and increased its nuclear localization. Consequently, lactylated Foxp3 interacted with IRF3 to enhance IL-10 expression and secretion, thereby augmenting Treg immunosuppressive function.

These findings address a key gap in our understanding of how Tregs adapt to the lactate-rich tumor microenvironment. The enrichment of Tregs in solid tumors and its correlation with poor patient prognosis have been well-documented clinically, yet the direct mechanisms linking high lactate concentrations in the TME to Treg functional regulation remain incompletely understood. The discovery of lysine lactylation has directly linked cellular metabolism to gene expression regulation, opening new avenues for understanding how metabolites shape immune cell function in the TME.

Previous studies have reported that MCT1-mediated lactate uptake promotes Treg function [36], and the upregulation of MCT1 in Tregs has been observed in some tumors. Our single-cell transcriptomic analysis of lung and melanoma tumor-infiltrating Tregs revealed that MCT4 upregulation was more pronounced and exhibited a distinct expression pattern compared with Th17 subsets. However, the GSE152022 dataset analyzed in Figure 1A,B contained a relatively low number of Th17 cells, leading to undersampling. Consequently, the observed differences should be interpreted with caution as they may not fully reflect the true biological effects due to these data constraints. The precise expression pattern and function role of MCT4 in Th17 cells warrant dedicated investigation. Under healthy or non-pathological conditions, a fine balance exists between Th17 and Treg cells, jointly maintaining immune homeostasis. Lactate treatment of Th17 cells increases genome-wide H3K18la levels and enhances IL-2 signaling, promoting the conversion of Th17 toward a Treg phenotype [64]. However, other studies have reported that lactate promotes the expression of Th17-related genes such as *Runx1*, *Tlr4*, *Il2*, and *Il4* via Ikzf1 K164 lactylation, thereby facilitating Th17 differentiation [68]. Th17 cells exhibit a dual role in tumor immunity: depending on the tumor microenvironment, they can either promote antitumor immunity or contribute to tumor progression and angiogenesis [69,70,71]. Whether differential MCT4 expression affects the Th17–Treg balance in tumors requires further investigation.

In vitro and in vivo studies showed that MCT1 and MCT4 display different expression kinetics in Tregs: MCT1 follows a “fast up, fast down” pattern, whereas MCT4 exhibits a “slow up, slow down” pattern, suggesting that MCT4 may play a more sustained role in chronic high-lactate environments. Although both MCT1 and MCT4 are bidirectional transporters [72,73], due to differences in their expression profiles and cellular metabolic states, MCT1 is typically considered to mediate lactate uptake, whereas MCT4 is primarily associated with lactate efflux. Using Treg-specific *Slc16a3* cKO mice, combined with ELISA and pH probe assays, we demonstrate for the first time that MCT4 non-redundantly mediates lactate uptake in Tregs. NaLa significantly promoted Treg stability and immunosuppressive function independently of TGF-β signaling, and this effect was dependent on MCT4 expression, indicating that MCT4-mediated lactate uptake is critical for Treg adaptation to high-lactate environments. Nevertheless, considering the established role of MCT4 as a lactate exporter, future studies using direct metabolic flux analysis (e.g., isotope tracing) are warranted to provide definitive kinetic evidence for lactate uptake by MCT4 in Tregs.

Lactylation provides a molecular basis for the direct regulation of protein function by metabolites. Our finding that NaLa treatment did not significantly alter Foxp3 mRNA expression or the H3K18 lactylation levels in Tregs suggests that NaLa regulates Foxp3 primarily at the protein level. Analysis of Foxp3 post-translational modifications identified multiple lactylation sites, with K277 as a key functional residue. Lactylated Foxp3 resisted ubiquitin-mediated degradation, thereby enhancing its stability and transcriptional regulatory function. Notably, it has been reported that USP21-mediated deubiquitination at K277 inhibits proteasomal degradation of Foxp3 [74,75,76]; however, the relevant E3 ubiquitin ligase and its regulatory mechanisms warrant further investigation.

Screening of downstream immunosuppressive molecules in Tregs identified IL-10 as a key functional mediator of NaLa effects. In vitro suppression assays and cKO mouse tumor models confirmed that NaLa enhances Treg immunosuppressive function by promoting IL-10 secretion. Mechanistically, NaLa promoted Foxp3 nuclear localization and enhanced the interaction between lactylated Foxp3 and IRF3, whereas the interaction between Foxp3 and IRF4 remained unaffected. Co-overexpression of FOXP3 and IRF3 significantly enhanced *Il10* promoter activity. While the IRF family members are known to play a pivotal role in *Il10* transcriptional regulation, whether IRF3 directly binds the *Il10* promoter and regulates its transcription in Tregs warrants further validation via chromatin immunoprecipitation assays. Furthermore, based on our current findings, we cannot exclude the potential contribution of epigenetic modifications, such as DNA methylation, histone acetylation, or histone lactylation to *Il10* expression. Additionally, the interplay between Foxp3 and other transcription factors, such as Blimp1, also remains to be explored. Tumor model studies in cKO mice revealed that MCT4 deficiency in Tregs leads to significant remodeling of the tumor immune microenvironment: reduced Treg infiltration and suppressive function, accompanied by increased CD8^+^ T cell infiltration and enhanced effector function. Using female heterozygous cKO mice, we directly compared the infiltration capacity of WT and MCT4-deficient Tregs within the same TME, confirming that MCT4 deficiency itself, rather than inter-individual differences in the TME, is responsible for reduced Treg infiltration. Treg infiltration in tumor tissues is regulated by multiple factors including apoptosis, proliferation, chemotaxis, induction, and stability. Through systematic investigation, we identified that the compromised stability of MCT4-deficient Tregs is the core mechanism underlying reduced tumor infiltration. Tregs exhibit a degree of phenotypic instability under physiological conditions [77,78], which can be exacerbated under inflammatory or stress conditions, manifesting as loss of Foxp3 expression and reduced suppressive function [79]. Although the TME is characterized by immunosuppression with multiple factors favoring Treg induction and function, it also imposes substantial environmental selection pressure on infiltrating Tregs. Our study demonstrates that the upregulation of MCT4 expression and subsequent lactate uptake represent an important adaptive mechanism by which tumor-infiltrating Tregs maintain their stability and suppressive function. Currently, therapeutic strategies targeting MCT1 are under preclinical and clinical investigation [80,81]. The upregulation of MCT4 in Tregs within high-lactate environments such as tumors positions MCT4 as a promising selective therapeutic target.

In conclusion, this study establishes the lactate–MCT4–Foxp3 lactylation axis as a critical regulator of Treg stability and functional adaptation in the tumor microenvironment, and identifies MCT4 as a promising therapeutic target for Treg-directed cancer immunotherapy. By demonstrating that MCT4-mediated lactate uptake stabilizes Foxp3 protein and enhances Treg suppressive function, our findings provide a mechanistic framework for targeting lactate metabolism to modulate Treg function in cancer.

## 4. Materials and Methods

### 4.1. Mice

C57BL/6 and CD45.1 mice were purchased from the Department of Laboratory Animal Science of Peking University Health Science Center. *Rag1^−/−^* mice were purchased from Vital River Laboratory Animal Technology (Beijing, China). *Foxp3*-EGFP and *Rosa26^tdTomato^* reporter mice were kindly provided by Prof. Qing Ge at the School of Basic Medical Sciences, Peking University. *Slc16a3^flox/flox^* and *Foxp3^YFP-Cre^* mice were purchased from Cyagen Biosciences (Suzhou, China). The *Slc16a3^flox/flox^* strain was generated by inserting loxP sites flanking exons 3–5 of the *Slc16a3* gene via CRISPR-mediated homologous recombination. Age-matched *Foxp3^YFP-Cre^Slc16a3^+/+^* mice were used as wild-type (WT) controls. All mice were housed and bred under specific pathogen-free (SPF) conditions in the Department of Immunology, Peking University Health Science Center, with a 12 h light/dark cycle at 22–23 °C. All animal experiments were approved by the Animal Welfare and Ethics Committee of Peking University Health Science Center (No. BCJB0082) and conducted in accordance with the 3R principles.

### 4.2. Cell Culture

The human embryonic kidney cell line HEK293T and mouse melanoma cell line B16 were purchased from ATCC (American Type Culture Collection, Manassas, VA, USA). HEK293T was cultured in DMEM (Gibco, Waltham, MA, USA) supplemented with 10% fetal bovine serum (FBS) and 1% penicillin–streptomycin; in some experiments, sodium lactate (Sigma-Aldrich, St. Louis, MO, USA) 20 mM. B16 and primary T cells were cultured in RPMI medium (Gibco) containing 10% FBS, 1% penicillin–streptomycin, and 50 μM β-mercaptoethanol; primary T cells were cultured in U-bottom 96-well plates pre-coated with 2 μg/mL anti-mouse CD3 antibody (Biolegend, San Diego, CA, USA) overnight at 4 °C, with 1 μg/mL soluble anti-mouse CD28 antibody (Biolegend) added to the culture medium. Polarization conditions were as follows: Th0, 10 ng/mL mouse IL-2 (Peprotech, Cranbury, NJ, USA); Th17, 20 ng/mL mouse IL-6 (Peprotech), 1 ng/mL mouse TGF-β1 (R&D), 10 μg/mL anti-mouse IFN-γ antibody (Biolegend) and 10 μg/mL anti-mouse IL-4 antibody (Biolegend); Treg, 10 ng/mL mouse IL-2 and 1–10 ng/mL mouse TGF-β1 depending on the experiment.

For Treg stability assays, sorted iTreg cells were rested for 48 h and then restimulated with 0.5 μg/mL anti-mouse CD3 and 0.25 μg/mL anti-mouse CD28 antibodies with or without sodium lactate 10 mM. All cells were maintained at 37 °C with 5% CO_2_. In the Il10 transcription experiment, sorted iTregs cells were rested with 100 nM auronofin (MCE, Monmouth Junction, NJ, USA).

### 4.3. B16 Tumor Model

B16 cells were passaged 3–4 times after thawing, harvested at ~70% confluence, and resuspended in PBS at a concentration of 1 × 10^7^ cells/mL. Eight- to ten-week-old mice were shaved on the right flank and subcutaneously injected with 100 μL of the cell suspension. For *Rag1^−/−^* mice, 1 × 10^6^ naïve CD8^+^ T cells (activated with TCR stimulation for 4 h) and/or 1 × 10^6^ induced Tregs (after expansion and sorting) were adoptively transferred via tail vein injection on day 4 post-tumor inoculation. Tumor size was measured every two days starting from day 5 using a caliper, and tumor volume was calculated as (length × width^2^)/2. Mice were euthanized between days 15 and 20 post-inoculation when the maximum tumor volume approached 2000 mm^3^. Tumors were harvested, weighed, photographed, and processed into single-cell suspensions by mincing and passing through 100-μm cell strainers. Spleens and draining lymph nodes were similarly processed after red blood cell lysis. For tumor-infiltrating lymphocyte isolation, single-cell suspensions were subjected to density gradient centrifugation using mouse lymphocyte separation medium (Cytiva, Marlborough, MA, USA) at 2000 rpm with slow acceleration and deceleration.

### 4.4. Flow Cytometry

For surface staining, cells were washed with PBS, stained with live/dead dye (1:60, diluted in PBS) for 15 min at 4 °C in the dark, and then incubated with a cocktail of surface antibodies diluted in FACS buffer (RPMI medium containing 1% FBS) for 30 min on ice. After washing, cells were resuspended in FACS buffer and filtered through 200-μm mesh before acquisition. For intracellular transcription factor staining, cells were fixed with fixation/permeabilization buffer (1:3 ratio, Thermo Fisher, Waltham, MA, USA) for at least 45 min at 4 °C, permeabilized with 1× Perm Buffer for 10 min, and stained with intracellular antibodies diluted in Perm Buffer for 1 h at 4 °C. For pH staining, cells were incubated with pHrodo™ Red AM (Thermo Fisher) in RPMI basal medium for 30 min at 37 °C, washed, and resuspended in RPMI for acquisition. Proper isotype antibodies were used as the control. All antibodies and dyes used are listed in Supplementary Table S1.

### 4.5. Treg Suppression Assay

Naïve CD4^+^ T cells isolated from CD45.2^+^ *Foxp3*-EGFP mouse spleens were induced into Tregs for 3 days. Tregs were pretreated with 10 mM NaLa for 24 h–48 h and sorted by flow cytometry to achieve >95% purity. In some experiment, iTregs cells were also pre-treated with auranofin (100 nM) for 48 h. On the day of sorting, CD45.1^+^ naïve CD4^+^ T cells were isolated from mouse spleens and labeled with CFSE to serve as responder T cells (Tresps). Irradiated splenocytes (2000 cGy) were used as feeder cells. Tregs and Tresps were co-cultured at various ratios with equal numbers of feeder cells and 1 μg/mL anti-mouse CD3 antibody. After 3 days, cells were stained with viability dye and antibodies against CD45.1 and CD45.2, and responder T cell proliferation was analyzed by flow cytometry. Where indicated, Foxp3 expression in the co-cultured Tregs was assessed using a Foxp3 fixation/permeabilization kit. The percentage of suppression was calculated as 100% − (proliferation percentage in the Treg group/proliferation percentage in the no-Treg group) × 100%.

### 4.6. Quantitative Real-Time RT-PCR

Total RNA was extracted using TRIzol (Life Technologies, Carlsbad, CA, USA), and the RNA concentration was measured with a NanoDrop spectrophotometer (Thermo Fisher). RNA with an A260/A280 ratio >1.8 was reverse-transcribed into cDNA using cDNA Synthesis SuperMix (TransGen Biotech, Beijing, China). Quantitative real-time PCR was performed on a PCRmax Eco 48 real-time PCR system (Illumina, San Diego, CA, USA) using FastStart Universal SYBR Green Master (Roche, Basel, Switzerland). Relative expression was calculated using the 2^−ΔΔCt^ method with the housekeeping gene as an internal control. Primer sequences are listed in Supplementary Table S2.

### 4.7. Western Blotting

Cells were lysed in lysis buffer containing 150 mM NaCl, 50 mM Tris-HCl (pH 7.4), 1 mM EDTA, 1% sodium deoxycholate, 1% Triton X-100, 0.1% SDS, and a protease and phosphatase inhibitor cocktail (Roche). Proteins were separated by SDS-PAGE and transferred onto nitrocellulose membranes. After blocking with BSA, membranes were incubated with primary antibodies (diluted 1:1000) overnight at 4 °C, followed by proper HRP-conjugated secondary antibodies (diluted 1:5000) for 1 h at room temperature. Protein bands were visualized using enhanced chemiluminescence substrate (Biodragon, Suzhou, China). All antibodies used are listed in Supplementary Table S3.

### 4.8. Mass Spectrometry

HEK293T cells transfected with the FOXP3-Flag plasmid using PEI were treated with 20 mM NaLa for 48 h and lysed in co-IP lysis buffer (20 mM Tris-HCl, pH 7.4, 150 mM NaCl, 1 mM EDTA, 1% Triton X-100) on ice for 1 h. Lysates were incubated with anti-Flag beads (Selleck, Houston, TX, USA) overnight at 4 °C, washed with co-IP wash buffer (20 mM Tris-HCl, pH 7.4, 500 mM NaCl, 1 mM EDTA, 1% Triton X-100), and eluted by heating in SDS loading buffer. Samples were resolved by SDS-PAGE, and gels were stained with Coomassie Brilliant Blue. The target protein bands were excised, subjected to in-gel digestion, and analyzed by liquid chromatography–tandem mass spectrometry (LC-MS/MS) using an EASY nLC 1200 system coupled to an LTQ Orbitrap Velos mass spectrometer. Raw data files were searched against the UniProt database using Mascot software (version 2.3.02). Peptide ion intensity spectra were processed and visualized using Thermo Xcalibur (version 2.2).

### 4.9. RNA-Seq Data Analysis

Tregs were sorted from the stability experiments and treated with 10 mM NaCl or NaLa for 48 h (three biological replicates per condition). Raw count data were log_2_-transformed after adding a pseudo-count of 1. Lowly expressed genes (counts <1 in <2 samples) were filtered out. Differential expression analysis between the NaLa and NaCl treated samples was performed using limma with a linear model (condition as factor). Genes with |log_2_ fold change| > log_2_(2) and adjusted *p*-value < 0.05 were considered significant. A Treg immunosuppressive gene set (including *Foxp3*, *Il2ra*, *Pdcd1*, *Ctla4*, *Entpd1*, *Nt5e*, *Il10*, *Il12a*, *Ebi3*, *Gzmb*, *Tnfrsf18*, *Icos*) was compiled. Heatmaps and PCA plots were generated using pheatmap (version 1.0.13) and ggplot2 (version 4.0.2).

### 4.10. Analysis of Single Cell RNA-Sequencing Data

All data (GSE126184 and GSE152022) were acquired from the Gene Expression Omnibus (https://www.ncbi.nlm.nih.gov/geo/, accessed on 5 September 2023).

Single-cell RNA-Seq data were analyzed using the Seurat R package (version 4.3.0) with default parameters unless otherwise specified. Samples were read into Seurat objects, and low-quality cells were filtered using the criteria: nFeature_RNA > 200 and <4000, and percentage of mitochondrial genes < 15%. Gene expression counts were normalized using the LogNormalize method. Highly variable features (*n* = 2000) were identified using the vst method, and all genes were scaled using the ScaleData function. PCA was performed on the scaled highly variable genes. Batch effects across samples were corrected using the RunHarmony function. Uniform manifold approximation and projection (UMAP) was applied for non-linear dimensionality reduction. Cells were clustered using the FindNeighbors and FindClusters functions (resolution = 1.5) based on the first 20 principal components. Cluster-specific marker genes were identified using the FindAllMarkers function. Th17 and Treg cell populations were defined based on marker gene expression profiles and used for downstream analysis.

### 4.11. Data Processing

Statistical analyses were performed using GraphPad Prism 9.0.0. Comparisons between two groups were analyzed using the two-tailed unpaired Student’s *t*-test. Comparisons among multiple groups were analyzed using one-way or two-way ANOVA (GraphPad Prism 9.0.0). All data are presented as the mean ± standard deviation (SD). A *p* value < 0.05 was considered statistically significant (* *p* < 0.05, ** *p* < 0.01, *** *p* < 0.001, ns, not significant).

## Data Availability

All relevant data supporting the findings of this study are included within the article. Further inquiries can be directed to the corresponding author.

## Supplementary Materials

The following supporting information can be downloaded at: https://www.mdpi.com/article/10.3390/ijms27104619/s1.

## Abbreviations

The following abbreviations are used in this manuscript:

AARS	Alanyl-tRNA synthetase
ATP	Adenosine triphosphate
CCR	C-C chemokine receptor
CFSE	Carboxyfluorescein succinimidyl ester
cKO	Conditional knockout
CTLA-4	Cytotoxic T-lymphocyte-associated protein 4
DC	Dendritic cell
ELISA	Enzyme linked immunosorbent assay
Foxp3	Forehead box p3
HDAC	Histone deacetylase
HLa	Lactate acid
ICOS	Inducible T cell co-stimulator
IL	Interleukin
IRF	Interferon regulatory factor
LDHA	Lactate dehydrogenase
MCT	Monocarboxylate transporter
NAD	Nicotinamide adenine dinucleotide
NaLa	Sodium lactate
NFAT1	Nuclear factor of activated T cells 1
PD-1	Programmed cell death protein 1
Tconv	Conventional T cells
TCR	T cell receptor
TGF-β	Transforming growth factor-β
TME	Tumor microenvironment
Tregs	Regulatory T cells
USP39	Ubiquitin-specific protease 39
YAP	Yes-associated protein
