# Supplementary material for: Lactate Uptake by MCT4 Facilitates Stability and Suppressive Function of Tumor-Infiltrating Regulatory T Cells by Promoting Foxp3 Lactylation
Source: Int J Mol Sci. 2026 May 21;27(10):4619. doi: 10.3390/ijms27104619 (PMC13207139; doi:10.3390/ijms27104619)
Supplement: Supplementary file 1 [file ijms-27-04619-s001.zip › ijms-4266014-supplementary.pdf]

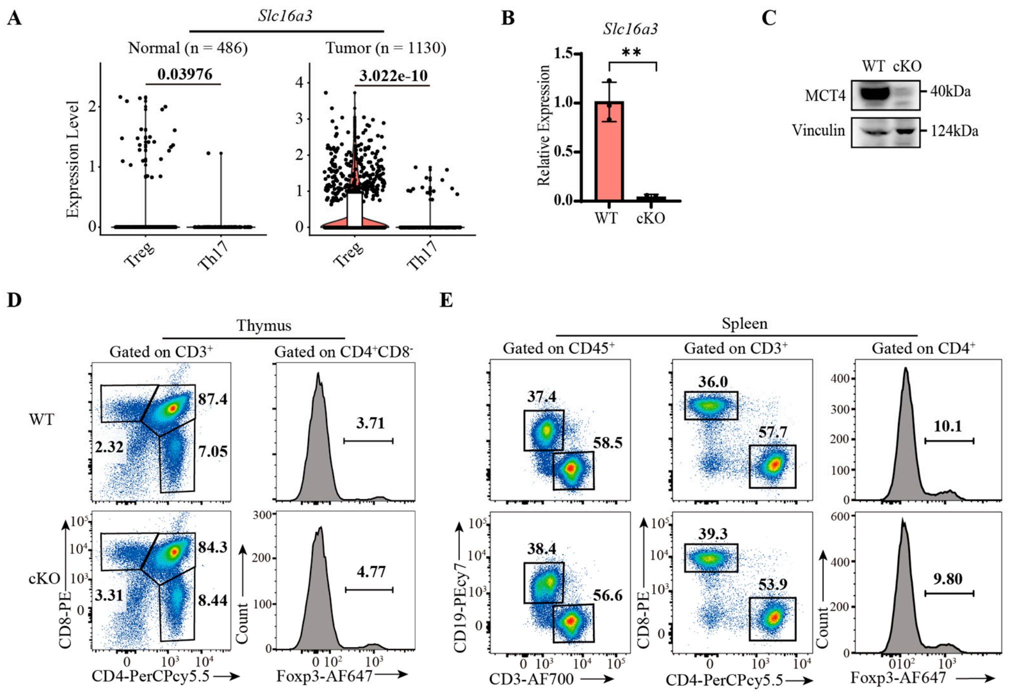

Figure S1. MCT4 mediates lactate uptake in tumor-infiltrated Tregs. (A) Direct comparison of *Slc16a3* expression levels in Treg and Th17 cells from normal and tumor tissues in the GSE152022 dataset. The Wilcoxon rank-sum test was performed, and the resulting *p*-values are shown above the boxplots. "n" indicates the number of cells analyzed from normal or tumor tissues, respectively. (B, C) Naive $CD4^+$ T cells from WT and cKO mice were induced to express Foxp3 *in vitro*, and YFP^+ cells were sorted by flow cytometry. Knockout efficiency was verified at the mRNA (B, $n = 3$) and protein (C) levels. (D, E) Thymus (D) and spleen (E) from WT and cKO mice were analyzed by flow cytometry to assess the proportions of $CD3^+$, $CD4^+$, $CD8^+$ T cells, and Tregs. Statistical analysis was performed using Student's t-test. $^{**}p < 0.01$.

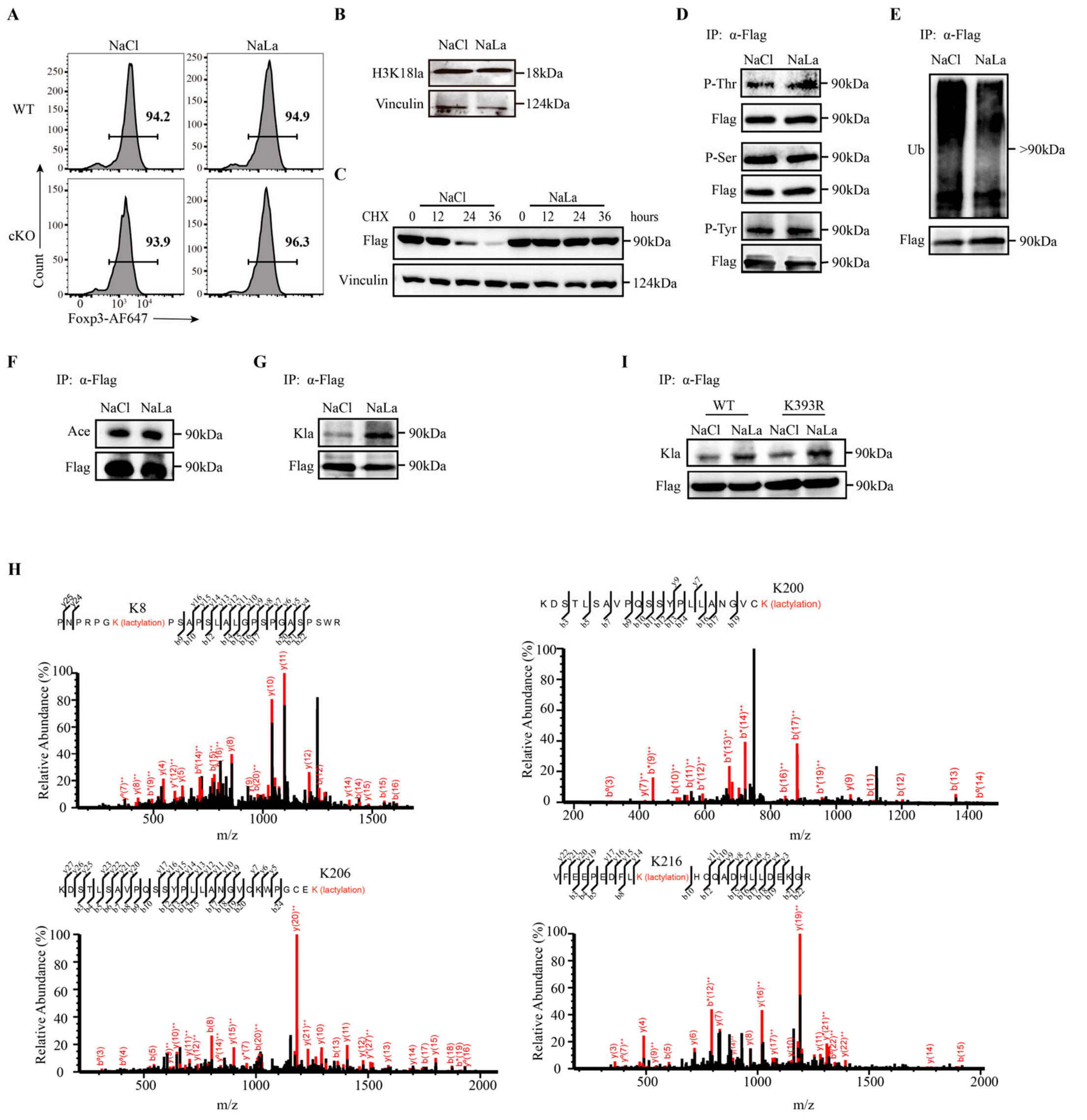

Figure S2. Uptake of lactate by MCT4 serves to enhance Foxp3 stability via K277 lactylation. (A) Purity of sorted Tregs used in *in vivo* stability experiments. (B) H3K18 lactylation levels in Tregs from stability experiments. (C) Western blotting analysis of HEK293T cells overexpressing FOXP3-Flag treated with 50 μmol/L CHX and 20 mM NaCl or NaLa for the indicated time points. (D-G) HEK293T cells overexpressing FOXP3-GFP-Flag were treated with 20 mM NaCl or NaLa for 48 h and subjected to immunoprecipitation-western blotting analysis using anti-Flag antibody. P, phosphorylation; Thr, threonine; Ser, serine; Tyr, tyrosine; Ub, ubiquitination; Ace, acetylation; Kla, lactylation. (H) Mass spectrometry analysis of FOXP3 K8, K200, K206, and K216 lactylation. "*" indicates a neutral loss during fragmentation of the precursor ion. (I) HEK293T cells overexpressing WT or K393R FOXP3-GFP-Flag were treated with 20 mM NaLa for 48 h and subjected to immunoprecipitation-western blotting analysis using anti-Flag antibody. WT, wild-type FOXP3; K393R, FOXP3 K393R mutant. Kla, lactylation.

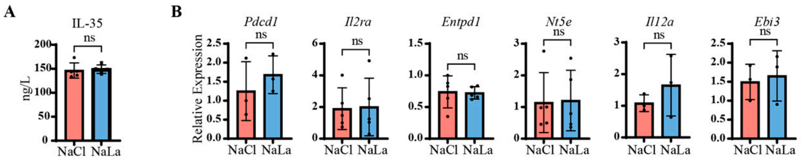

Figure S3. Lactylated Foxp3 promotes IL-10 secretion, enhancing Treg suppressive function. (A) ELISA measurement of IL-35 in Treg culture supernatants from stability experiments. $n = 5$. (B) *Pdcd1*, *Il2ra*, *Entpd1*, *Nt5e*, *Il12a* and *Ebi3* mRNA expression in Tregs from stability experiments. For *Pdcd1*, *Il12a*, and *Ebi3*, $n = 3$; for *Il2ra*, *Entpd1*, and *Nt5e*, $n = 5$. Statistical analysis was performed using Student's t-test. ns, $p > 0.05$.

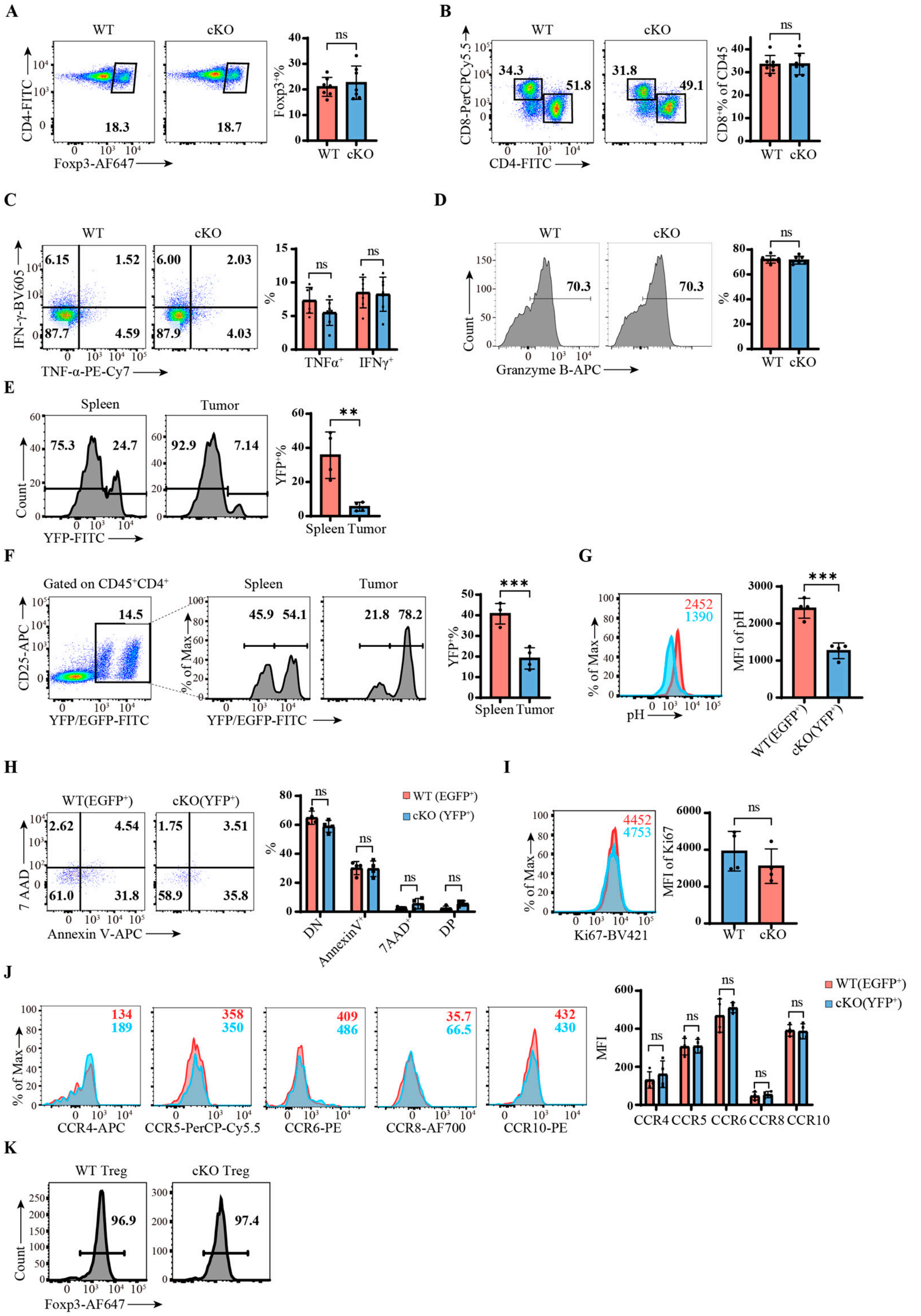

Figure S4. Lactate uptake by MCT4 is essential for maintaining tumor-infiltrating Treg stability. (A–D)

B16 melanoma model in *Slc16a3*$^{flox/flox}$*Foxp3*$^{YFP\text{-}cre}$ (cKO) mice. $n = 7$. (A) Proportion of Tregs in draining lymph nodes. (B) Proportion of $CD8^+$ T cells in draining lymph nodes. (C, D) Cytokine production by $CD8^+$ T cells in draining lymph nodes. (E) Proportion of YFP^+ and YFP^- cells among $CD4^+CD25^+$ tumor-infiltrating Tregs from *Slc16a3*$^{flox/flox}$*Foxp3*$^{YFP\text{-}cre/+}$ mice bearing B16 melanomas. $n = 4$. (F) Proportion of YFP^+ and $EGFP^+$ cells among $CD45^+CD4^+FITC^+$ tumor-infiltrating Tregs from *Slc16a3*$^{flox/flox}$*Foxp3*$^{YFP\text{-}cre/EGFP}$ mice bearing B16 melanomas. Cells with higher fluorescence intensity are $EGFP^+$; cells with intermediate fluorescence intensity are YFP^+; both are collectively referred to as $FITC^+$. $n = 4$. (G) pH probe staining of tumor-infiltrating Tregs from *Slc16a3*$^{flox/flox}$*Foxp3*$^{YFP\text{-}cre/EGFP}$ mice. $n = 4$. (H) Annexin V and 7-AAD staining of tumor-infiltrating Tregs from *Slc16a3*$^{flox/flox}$*Foxp3*$^{YFP\text{-}cre/EGFP}$ mice. DN, double negative; DP, double positive. $n = 4$. (I) Ki67 expression in tumor-infiltrating Tregs from *Slc16a3*$^{flox/flox}$*Foxp3*$^{YFP\text{-}cre}$ cKO mice and *Slc16a3*$^{+/+}$*Foxp3*$^{YFP\text{-}cre}$ WT mice. $n = 4$. (J) Chemokine receptor expression in tumor-infiltrating Tregs from *Slc16a3*$^{flox/flox}$*Foxp3*$^{YFP\text{-}cre/EGFP}$ mice. $n = 4$. (K) Foxp3 expression purity of sorted Tregs used in *Rag1*$^{-/-}$ mouse adoptive transfer experiments. Statistical analysis was performed using Student's t-test. $^{**}p < 0.01$, $^{***}p < 0.001$, ns, $p > 0.05$.

Table S1. Antibodies for Flow Cytometry

Antibody	Cas.	Company
Anti-mouse CD45.1-APC	110714	Biolegend
Anti-mouse CD45.2-PerCP	109826	Biolegend
Anti-mouse CD45-Percp-cy5.5	103130	Biolegend
Anti-mouse CD3e-AF700	100216	Biolegend
Anti-mouse CD4-PerCPcy5.5	550954	BD Bioscience
Anti-mouse CD4-FITC	100406	BD Bioscience
Anti-mouse CD4-APC-cy7	100414	Biolegend
Anti-mouse CD8a-FITC	100706	Biolegend
Anti-mouse CD8a-PerCP	100732	Biolegend
Anti-mouse Foxp3-AF647	320014	Biolegend
Anti-mouse CD73-PE	127206	Biolegend
Anti-mouse CD39-APC	143810	Biolegend
Anti-mouse CD25-PEcy7	113711	Biolegend
Anti-mouse PD-1-APCcy7	135224	Biolegend
Anti-mouse CTLA-4-BV421	106312	Biolegend
Anti-mouse ICOS-AF700	313528	Biolegend
Anti-mouse GITR-PE	126310	Biolegend
Anti-mouse IL-10-PE	505008	Biolegend
Anti-mouse IFN-γ-BV605	505810	Biolegend
Anti-mouse Granzyme B-APC	396408	Biolegend
Anti-mouse TNF-α-PEcy7	506324	Biolegend
Anti-mouse Ki67-BV421	350506	Biolegend
Anti-mouse MCT4-PE	sc-376101 PE	Santa Cruz
Anti-mouse CCR4-APC	G1353	Selleck
Anti-mouse CCR5-PerCPcy5.5	107016	Biolegend
Anti-mouse CCR6-PE	129804	Biolegend
Anti-mouse CCR8-AF700	150334	Biolegend
Anti-mouse CCR10-PE	FAB2815P	R&D
APC anti-Annexin V	640920	Biolegend
7AAD	00-6993-50	eBioscience
CFSE	C34570	Thermo Fisher
LIVE/DEAD FIX AQUA	L34965	Biolegend

Table S2. Primer Sequences for qPCR

Primer	Sequence (5′-3′)
β-actin	F: GGCTGTATTCCCCTCCATCG<br>R: CCAGTTGGTAACAATGCCATGT
Pdcd1	F: CGGTTTCAAGGCATGGTCATTGG<br>R: TCAGAGTGTCGTCCTTGCTTCC
Il2ra	F: GCGTTGCTTAGGAAACTCCTGG<br>R: GCATAGACTGTGTTGGCTTCTGC
Entpd1	F: CTGGACAAGAGGAAGGTGCCTA<br>R: GACTGTCTGAGATGAGGCTTAGC
Nt5e	F: CGCTCAGAAAGTTCGAGGTGTG<br>R: CGCAGGCACTTCTTTGGAAGGT
Il10	F: AGCCTTATCGGAAATGATCCAGT<br>R: GGCCTTGTAGACACCTTGGT
Il12a	F: CTGTGCCTTGGTAGCATCTATG<br>R: GCAGAGTCTCGCCATTATGATTC
Ebi3	F: CTCTCAAGTACCGACTCCGCTA<br>R: CTGAGCTGACACCTGGATGCAA
Slc2a1	F: ATGGATCCCAGCAGCAAG<br>R: CCAGTGTTATAGCCGAACTGC
Slc16a3	F: TCCATCCTGCTGGCTATGCTCT<br>R: CAGAAGGACGCAGCCACCATTC
Foxp3	F: TTCCTTCCCAGAGTTCTTCC<br>R: CTCAAATTCATCTACGGTCCA

Table S3. Antibody for Western Blotting

Antibody	Cas.	Company
Rabbit anti-Flag	14793S	CST
Mouse anti-MCT1	MA518288	Invitrogen
Rabbit anti-Vinculin	13901S	CST
Rabbit anti-Lamin B1	13435S	CST
Mouse anti-Myc	2276S	CST
Rabbit anti-IRF3	4302S	CST
Rabbit anti-MCT4	22787-1-AP	Proteintech
Rabbit anti-Acetyl Lysine	9681S	CST
Rabbit anti-p-Thr	PTM-705RM	PTM Biolabs
Rabbit anti-p-Ser	PC11993S	Abmart
Mouse anti-p-Tyr	9416S	CST
Rabbit anti-L-Lactyl Lysine	PTM-1401RM	PTM Biolabs
Rabbit anti-ubiquitin	43124S	CST
Rabbit anti-H3K18la	PTM-306RM	PTM Biolabs
Mouse anti-Foxp3	14-7979-82	Invitrogen
